# The Influence of Heat Treatment on the Mechanical Properties and Corrosion Resistance of the Ultrafine-Grained AA7075 Obtained by Hydrostatic Extrusion

**DOI:** 10.3390/ma15124343

**Published:** 2022-06-20

**Authors:** Marta Orłowska, Ewa Ura-Bińczyk, Lucjan Śnieżek, Paweł Skudniewski, Mariusz Kulczyk, Bogusława Adamczyk-Cieślak, Kamil Majchrowicz

**Affiliations:** 1 Faculty of Mechanical Engineering, Military University of Technology, Gen. S. Kaliskiego 2, 00-908 Warsaw, Poland; lucjan.sniezek@wat.edu.pl; 2 Faculty of Materials Science and Engineering, Warsaw University of Technology, Wołoska 141, 02-507 Warsaw, Poland; ewa.ura@pw.edu.pl (E.U.-B.); pskudniewski@gmail.com (P.S.); boguslawa.cieslak@pw.edu.pl (B.A.-C.); kamil.majchrowicz@pw.edu.pl (K.M.); 3 Institute of High Pressure Physics, Polish Academy of Sciences, Sokolowska 29/37 St., 01-142 Warsaw, Poland; mariusz.kulczyk@unipress.waw.pl

**Keywords:** 7075 aluminum alloy, hydrostatic extrusion, heat treatment, microstructure, mechanical properties, corrosion resistance

## Abstract

In this paper, the corrosion resistance and mechanical properties of the 7075 aluminum alloy are studied. The alloy was deformed by hydrostatic extrusion and then aged both naturally and artificially. Results are compared with those of coarse-grained material subjected to T6 heat treatment. The aim of the research is to find the optimal correlation between the mechanical properties and the corrosion resistance of the alloy. To this end, static tensile tests with subsequent fractography, open circuit potential, and potentiodynamic polarization tests in 0.05 M NaCl were conducted. Obtained results show that a combination of precipitate hardening and a deformed microstructure leads to increased mechanical strength with high anisotropy due to the presence of fibrous grains. Plastic deformation increases susceptibility to corrosion due to the increased number of grain boundaries, which act as paths along that corrosion propagates. However, further artificial aging incurs a positive effect on corrosion resistance due to changes in the chemical composition of the matrix as a result of the precipitation process.

## 1. Introduction

The 7XXX series of Al–Zn–Mg(–Cu) aluminum alloys (AAs) are highly important industrial materials, primarily due to their light weight, high strength, and good corrosion resistance. Such materials are commonly used in the construction of aircraft, among other uses. As a group of materials, the 7XXX alloys are particularly interesting because of the process of precipitation hardening, which causes a complex microstructure. Such a microstructure is composed of fine hardening precipitates and coarse intermetallic phases within the Al matrix [1]. For the 7XXX family, this includes Al_3_Fe, Al_2_CuMg, Al_2_Cu, Al_3_(FeCu), Al_7_Cu_2_Fe, or Mg_2_Si particles. The hardening precipitates are formed and distributed throughout the matrix via proper heat treatment: supersaturated solid solutioning followed by aging. The principal precipitation sequence that dominates hardening in most commercially used 7XXX alloys is presented in Equation (1):

SSSS∝ → GP zones → η′ → η(1)

where SSSSα represents a supersaturated solid solution, GP zones are Guinier–Preston zones, η′ is a metastable phase (with a Mg:Zn ratio in the range from 1:1 to 1:1.15), and η is a stable MgZn_2_ phase. MgZn_2_ plays the most significant role in the precipitate hardening process [2].

The mechanical properties of Al–Zn–Mg–Cu quaternary alloys are determined primarily by precipitation hardening, where the nanometer-scale precipitates act as pinning centers to prevent dislocation motion [3]. Hardening is achieved via the presence of microstructural obstacles that inhibit dislocation movement and improve the overall mechanical properties of the alloy [4]. Such obstacles are also grain boundaries. Reducing the grain size increases the fraction of grain boundaries within the material and correspondingly increases the mechanical strength of the material. The application of severe plastic deformation (SPD) [5,6] leads to grain formation and achieves an ultrafine-grained (UFG) microstructure, which substantially increases mechanical strength. Following SPD, 7XXX alloys exhibited an increase in tensile strength from 228 to 434 MPa [7]. However, the greatest increases in strength are obtained when SPD is combined with precipitate strengthening. The application of hydrostatic extrusion followed by aging increases the tensile strength of AA 7475 to 700 MPa [8]. As such, the optimal microstructure to improve the mechanical strength of 7XXX alloys has a fine grain size with a uniform dispersion of small hard particles to inhibit dislocation motion. 

Along with mechanical properties, corrosion resistance is also affected by both precipitates and grain boundaries. Nevertheless, there is no clear correlation, as corrosion resistance is influenced by various microstructural factors and also the environment in which the examination takes place. Therefore, determining the influence of one microstructural component on the corrosion resistance is challenging. Nevertheless, there are works devoted to these topics. In the case of grain size, susceptibility to corrosion may decrease [9]; in the case of AAs, it reduces cathodic kinetics, improves the results of mass-loss testing, and in some cases improves resistance to stress corrosion cracking [10]. Studies that suggest that corrosion resistance increases with decreasing grain size generally find that the refined microstructures are attributed to an increased readiness to passivate compared to coarse-grained (CG) materials due to a higher grain boundary density [11]. Further to grain refinement, SPD physically breaks down second-phase or intermetallic particles below a critical size, which subsequently prevents these second-phase particles from operating as efficient local cathodes and sites for the initiation of localized attack [9,12]. However, some works present an opposing view: corrosion rate may also increase as AA grain size increases [9,13]. In [14], friction stir processing was used to refine the microstructure of AA 6063. The smaller grains produced by such an approach were more susceptible to corrosion in 1 M HCl. Furthermore, Osorio et al. suggested that, when exposed to NaCl electrolytes, CG AAs showed better corrosion resistance than that of fine-grained AAs, as they had fewer corrosion initiation sites [15]. 

The purpose of this work is to correlate the changes in the microstructure caused by plastic deformation and subsequent heat treatment with the mechanical properties and corrosion resistance of the AA7075. However, in opposition to the previous works devoted to this topic, the approach of maintaining the size and distribution of intermetallic particles was undertaken. This is crucial in terms of corrosion resistance, as such particles are the places where corrosion attack initiates due to their electrochemical properties; therefore, their influence is the most significant. In the present study, due to preserving the size of the intermetallic particles, other structural components were investigated, i.e., grain boundaries and second-phase particles. As 7XXX AAs have a complex microstructure that is determined by thermomechanical processing, the primary goal of this study is to determine the optimal heat treatment parameters of ultrafine-grained AA 7075, obtained by hydrostatic extrusion. The aim of the study is to increase the mechanical strength of AA 7075 while maintaining its high corrosion resistance. 

## 2. Materials and Methods

The research subject is commercially available AA 7075, with its chemical composition given in Table 1, delivered in the form of rods with a diameter of ∅20. The manufacturer of the alloy was Kamensk Uralsky Metallurgical Works. For the purpose of this study, the samples underwent different combinations of heat treatment and SPD. The SPD method used was hydrostatic extrusion [16]. In this approach, the billet is located in the container and surrounded with a pressure transmitting medium. The piston compresses the medium until the billet starts to extrude through the die. The negligible friction between the billet and the die allows for the use of small die angles that ensure that the high deformation is homogeneous. The unique features of hydrostatic extrusion are three-axial compressive stresses within the billet and high strain rates greater than 10^4^ s^−1^. In comparison to standard SPD methods, as a means of grain refinement, this process requires significantly smaller total strain. In this study, hydrostatic extrusion was performed in a single step with a reduction in the sample diameter from ∅20 to ∅10. The equivalent strain of hydrostatic extrusion is given by Equation (2):

(2) $$\varepsilon =2ln\left(\frac{d^{\prime }}{d^{\prime \prime }}\right)$$

where *d*′ is the initial diameter of the rod, and *d*″ is the exit diameter. The equivalent strain following the single-step hydrostatic extrusion process that we used was approximately 1.4.

The following list of samples were examined:

- CG, a coarse-grained precipitation-strengthened sample used as a reference material, solution heat-treated with T6 aging following hydrostatic extrusion to maintain the size and location of intermetallic inclusions.
- HE, an ultrafine-grained sample naturally aged for 180 days following hydrostatic extrusion.
- HT1, an ultrafine-grained sample precipitation strengthened following hydrostatic extrusion (artificially aged at 100 °C for 24 h).
- HT2, an ultrafine-grained sample precipitation strengthened following hydrostatic extrusion (artificially aged at 120 °C for 24 h).

For the aging process, an SUP-30G furnace from WAMED Co. was used, which has very high certainty of maintaining the temperature, i.e., ±0.2 °C.

The microstructure and postcorrosion morphology of the samples were investigated using a Hitachi Su70 scanning electron microscope (SEM). The surface corrosion attack was observed with samples polarized up to 0.1 mA/cm^2^, and the postcorrosion morphology (the number and shape of pits) of the sample cross-sections was examined. The depth and morphology of the pits were evaluated on the longitudinal cross-section of the sample, oriented along the grains in the direction of hydrostatic extrusion). Energy-dispersive X-ray spectroscopy (EDS) was used to perform the elemental analysis and chemical characterization of the intermetallic particles present in the microstructure. Electron backscatter diffraction (EBSD) was used to examine the crystallographic orientation of grain and grain boundary characteristics. We determined the crystallographic orientations of the grains, the grain size, the fraction of high angle grain boundaries (HAGBs), and the fraction of low angle grain boundaries (LAGBs). The data collection step size was 250 µm.

For detailed microstructure characterization, a JEOL JEM-1200 transmission electron microscope (TEM) operating at 120 kV was used. TEM samples were sectioned along the transverse and longitudinal axes of the rods. Thin TEM foils were prepared by grinding slices down from a thickness of 1 mm into a thickness of 150 µm, and then electropolishing them using a solution of Struers electrolyte A2 at 5 °C and 25 V. To complement the TEM investigations, we also used X-ray diffraction (XRD). To correctly identify the visible precipitates on the TEM samples, XRD measurements were taken using a Bruker D8 Advance diffractometer with Cu Kα radiation. Specimens were rotated at 10 rpm during the diffraction recording. Diffraction lines were matched to the pattern using EVA V3.0 software.

The mechanical properties of the processed samples were determined by tensile tests. For each sample, five measurements were taken. Static tensile tests were carried out on flat minisamples [17,18] with cross sections of 0.6 × 0.8 mm and gauge lengths of 5 mm. The tests were performed at room temperature at an initial strain rate of 1 × 10^−3^ s^−1^, using digital image correlation for noncontact strain measurements. We determined quantitative average values for ultimate tensile strength (UTS), 0.2% offset yield strength (YS), and elongation at break (E_b_). Due to the size of the examined tensile test samples, measurements were taken from two directions, longitudinal and transverse, with respect to the extrusion direction. This approach allowed for the anisotropy of the mechanical properties to be examined. After the tensile tests, fractography was performed on an SEM.

Corrosion resistance was investigated via electrochemical experiments. Samples were subjected to cyclic polarization in potentiodynamic (PP) mode. Before each test, each sample was immersed in the prepared electrolytes for 10 min to stabilize the rest potential. Additionally, the change in open circuit potential (E_OCP_) was observed for 48 h to determine the behavior of the material in the corrosive environment over an extended period of time. To produce homogeneous, comparable surfaces and ensure the presence of a repeatable passivation layer, all samples were ground by silicon carbide water grinding abrasive paper, with grades of up to #4000, and then placed within a desiccator for 24 h prior to corrosion testing. All tests were performed at room temperature, with a corrosive medium of Cl– ions within an aqueous environment. To this end, a solution of 0.05 M NaCl using distilled water was prepared. A NOVA AutoLab PGSTAT302N potentiostat was used to control the potential between the working electrode (sample) and the Ag/AgCl reference electrode while measuring the current between the working electrode and the platinum sheet counter electrode. The surface of each sample was sealed in plastic, with a 20 mm^2^ area left exposed. Corrosion tests were conducted on the surfaces perpendicular to the hydrostatic extrusion direction. The cyclic PP with a 1 mV/s scan rate started from −0.05 V relative to the E_OCP_, and this was reversed when the current had reached a value of 0.3 mA. These measurements were repeated three times to ensure the reproducibility of the results. Average corrosion parameters, including corrosion potential (E_corr_), corrosion current density (i_corr_), and repassivation potential (E_rep_), were obtained from the curves.

## 3. Results

### 3.1. Microstructure

#### 3.1.1. SEM/EBSD

**Figure 1:**
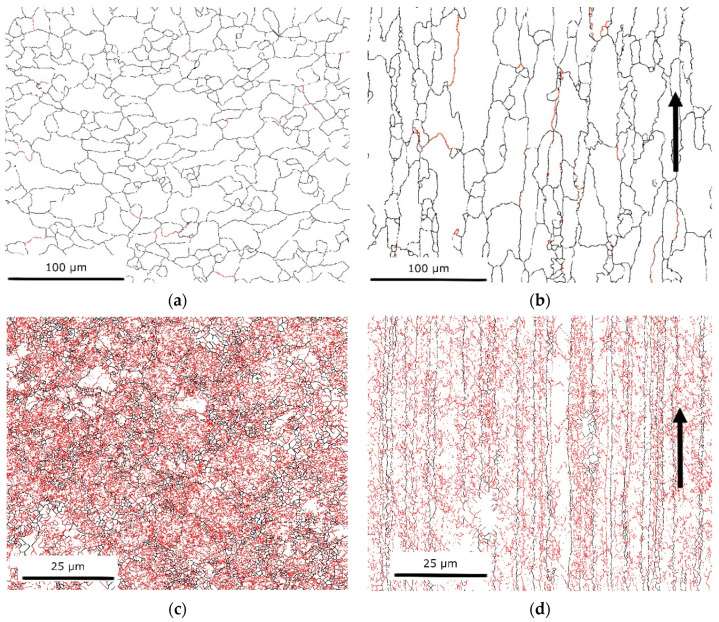
Grain boundary maps, with LAGBs shown in red, and HAGBs shown in black: (**a**) CG transverse plane, (**b**) CG longitudinal plane, (**c**) HE transverse plane, and (**d**) HE longitudinal plane; black arrows indicate the direction of the hydrostatic extrusion.

Maps of the grain boundaries of CG and HE samples are presented in Figure 1. Both the transverse and longitudinal planes are shown with respect to the extrusion direction. For the latter, the extrusion direction is indicated by black arrows. There are noticeable differences between the samples. In the transverse plane, the CG sample contained a large amount of HAGBs, with a much smaller number of LAGBs distributed throughout. The grains were equiaxial with an average grain size of 17.1 μm. The largest grain exceeded 40 μm in size. The hydrostatic extrusion process caused significant grain refinement, resulting in an average grain size of 1.3 μm (including subgrains) for the HE sample. This difference was primarily caused by a considerable increase in the density of LAGBs: the CG sample contained 5.56% LAGBs, while the HE sample contained 76%. LAGBs are predominantly located in the grain interiors, creating a network of subgrains surrounded by the HAGBs which delineate and separate each grain.

In the longitudinal plane, HAGBs lay parallel with one another in accordance with the extrusion direction, creating strongly elongated grains. The heat treatment of the CG sample resulted in a larger average grain size of 17.6 μm. Nevertheless, the elongated shape was preserved. Within the grains, a small number of LAGBs were observed. For the HE sample, a network of LAGBs existed within the interiors of significantly elongated grains, which were separated by the HAGBs. LAGBs propagated both perpendicular and parallel to the hydrostatic extrusion direction. The average grain size of the HE sample within the longitudinal plane was 1.7 μm. The length of the lamellas defined by the HAGBs exceeded 75 μm, while their maximal thickness did not exceed 10 μm.

**Figure 2:**
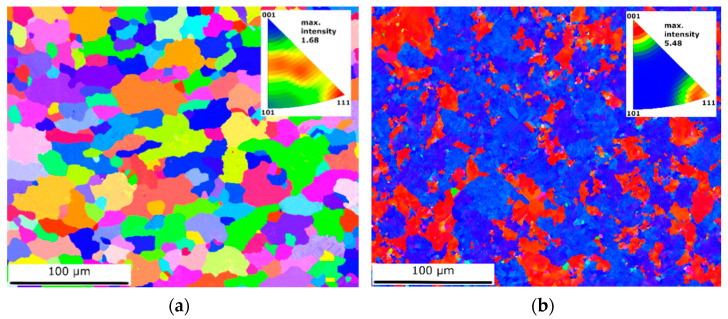
Orientation maps of (**a**) CG and (**b**) HE samples.

The crystallographic orientation of the grains was also analyzed. Figure 2 shows grain orientation maps of the transverse sample planes. The inverse pole figures for each sample are shown as insets. A comparison of these maps shows that a strong texture developed following hydrostatic extrusion. For the CG sample, the grain orientation was scattered, with preferred orientations of <111>, <112>, and <012>. However, the maximal measured intensity was only 1.68. For the HE sample, two distinct dominant crystallographic orientations could be identified: <001> and <111>. Moreover, the maximal intensity of 5.48 was larger than that of the CG sample.

#### 3.1.2. XRD

**Figure 3:**
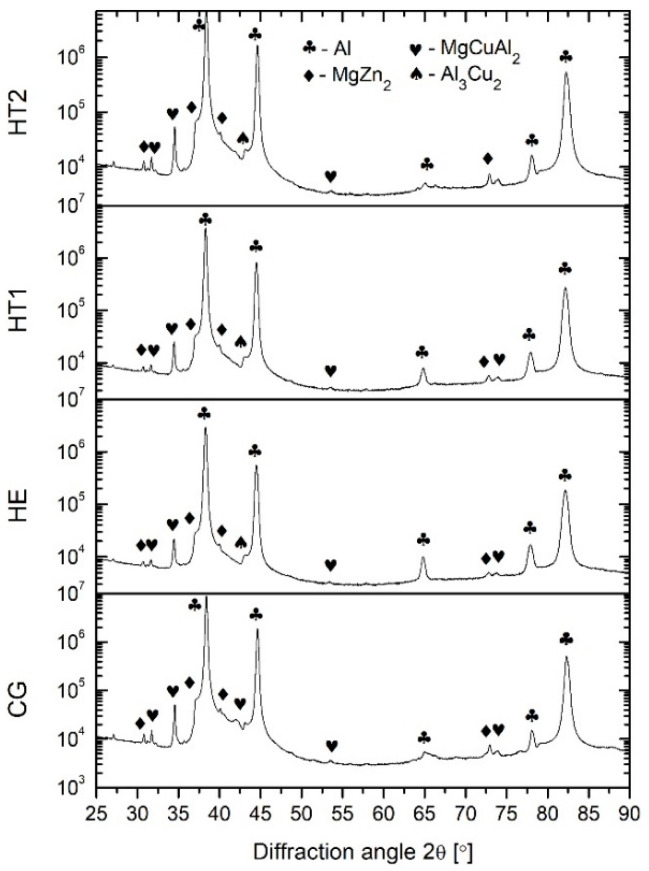
XRD patterns of examined samples (PDF no. of identified phases: PDF 00-004-0787—Al, PDF 01-073-5874—MgCuAl_2_, PDF 00-034-0457—MgZn_2_, PDF 01-071-5716—Al_3_Cu_2_).

Samples were analyzed using XRD to identify the phases. The corresponding XRD patterns are presented in Figure 3. XRD peaks could be identified mainly as ∝ Al solid solution (labeled as Al), η-precipitates (labeled as MgZn_2_), and also some peaks as MgCuAl_2_ and Al_3_Cu_2_. In general, the XRD peaks were slightly stronger for the CG sample than those for the other samples. However, the XRD diffraction peaks originating from the η precipitates were weak for all samples. Following hydrostatic extrusion, a strong texture caused the incomplete detection of peaks originating from the Al solid solution. The nonequilibrium phase (η′) was also detected, but the extremely small size of the η′-phase precipitates resulted in diminished XRD peaks and significantly complicated their proper description. Furthermore, for samples that had undergone hydrostatic extrusion, XRD graphs were very similar, with the same identified constituents.

#### 3.1.3. TEM

**Figure 4:**
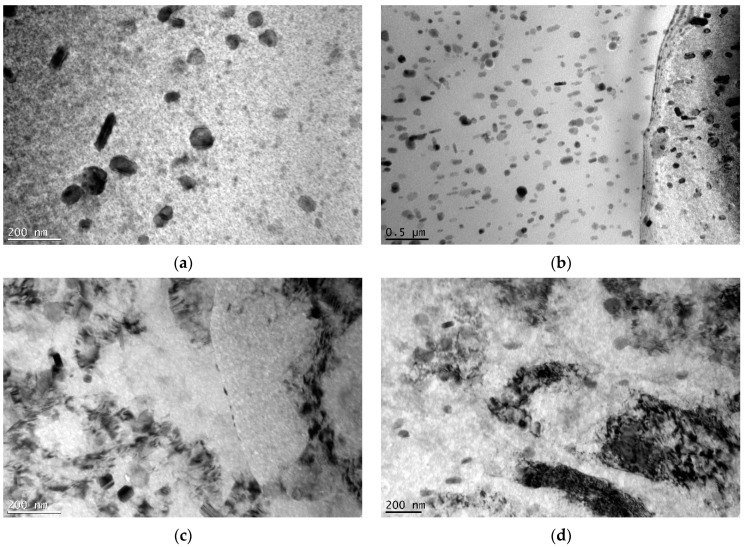
TEM micrographs of the samples: (**a**) transverse plane of CG, (**b**) longitudinal plane of CG, (**c**) transverse plane of HE, and (**d**) longitudinal plane of HE.

TEM bright-field images of the CG sample are presented in Figure 4a,b. The microstructure of the CG sample consisted of equiaxed grains with finely dispersed η-precipitates (MgZn_2_). The majority of such precipitates were located within the grains; a small fraction were located at grain boundaries. Much smaller nonequilibrium precipitates (η′) were homogeneously distributed in the grain interiors. These precipitates are the blurry disc- or rod-shaped dots in the background of the images. Figure 4c,d show that the microstructure of the naturally aged HE sample was significantly deformed. The dark grain areas likely indicate the presence of a high density of dislocations. This suggests that the microstructure obtained via the hydrostatic extrusion process was highly deformed, with significant stress applied to the material that correspondingly resulted in the generation of dislocations. Compared to the CG sample, the number and size of η precipitates visible within the grains were significantly reduced for the HE sample. However, those that were observed were homogeneously distributed within the microstructure. Moreover, the precipitates were also present at the grain boundaries. Fine metastable precipitates (η′) could also be observed within the grains.

**Figure 5:**
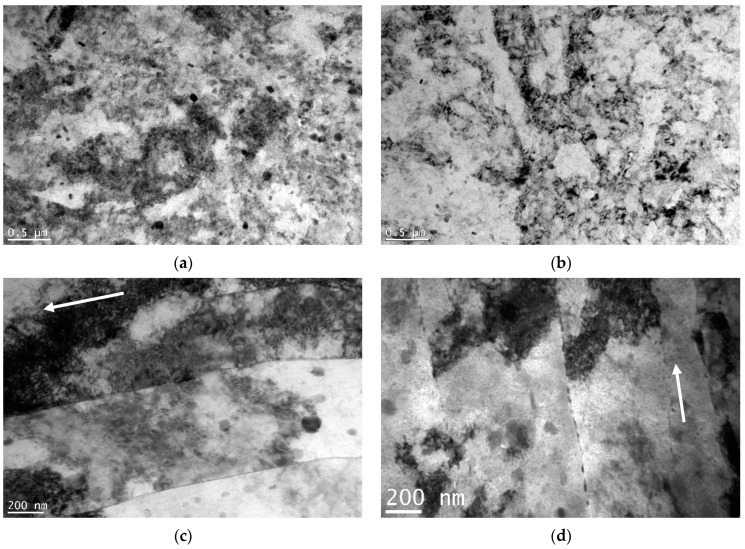
TEM micrographs of the samples: (**a**) HT1 transverse plane, (**b**) HT2 transverse plane, (**c**) HT1 longitudinal plane, and (**d**) HT2 longitudinal plane; white arrows indicate the direction of the hydrostatic extrusion.

TEM micrographs of samples HT1 and HT2 are presented in Figure 5. Regarding the HE sample, a deformed microstructure with a high density of dislocations could be observed. Elongated grains were visible within the longitudinal plane, with precipitates formed both within the grains and on the grain boundaries. The grains were elongated along the extrusion direction, which is marked by white arrows. The η precipitates of the HT1 and HT2 samples were much smaller than those of the CG sample, but larger than those of the HE sample, indicating that an increased aging temperature increases the size of η precipitates within the microstructure following hydrostatic extrusion. Moreover, the numbers of precipitates in the HT1 and HT2 samples were increased in comparison to those in the HE sample. As the aging temperature increased, the size of the precipitates at the grain boundaries increased. The HT1 sample had slightly larger η precipitates than those of the HT2 sample. For both samples, η′ precipitates were also visible.

#### 3.1.4. SEM/EDS

**Figure 6:**
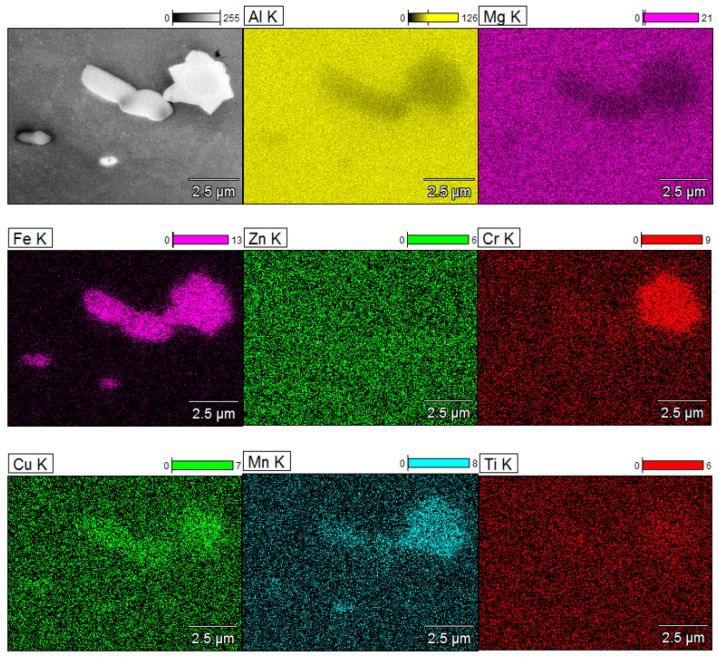
EDS mapping results of inclusions within AA 7075.

The intermetallic particles were examined using SEM/EDS. Figure 6 presents the EDS mapping results, showing the chemical composition of the intermetallic particles (inclusions) within the microstructure. As the CG sample was heat-treated following hydrostatic extrusion to obtain a coarse-grained microstructure, there was no noticeable difference between each sample in the shape and size of the particles. Results demonstrate that the majority of the analyzed particles consisted of Al, Cu, and Fe, with a smaller amount of Mn and a very small amount of Cr. The particles were comparatively large and irregularly shaped, with sizes of 1–10 μm. The particles were formed during alloy solidification and did not dissolve during subsequent thermomechanical processing [19]. From the literature, the particles that occur most commonly in AA 7075 and correspond with the compounds that we identified are Al_7_Cu_2_Fe, Al_2_CuMg, Al_2_Cu, and Al_3_Fe [20,21]. Compounds that contain Cu and Fe are nobler than pure Al, resulting in the dissolution of more active matrix and leading to the appearance of rings around a nearly intact particle or particle colony [22]. This phenomenon was observed later, during the postcorrosion damage evaluation.

### 3.2. Mechanical Properties

**Figure 7:**
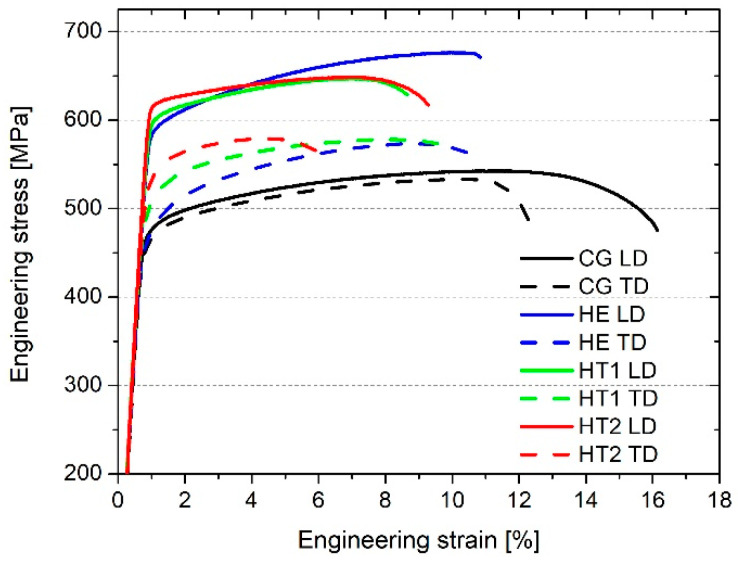
Representative stress–strain curves of the samples.

**Figure 8:**
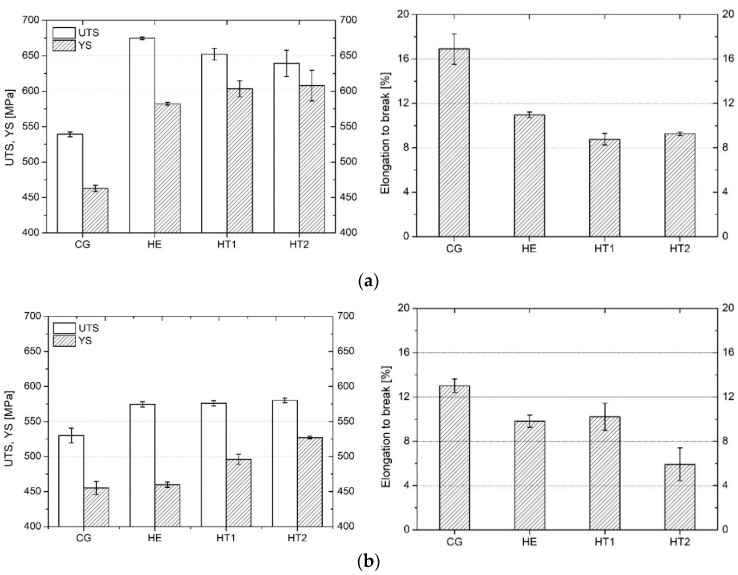
Values of UTS, YS, and E_b_ for each sample, measured in the (**a**) longitudinal and (**b**) transverse directions with respect to the extrusion direction.

The representative stress–strain curves are shown in Figure 7, while the average results of YS, UTS, and E_b_ tests are presented in Figure 8. The values of UTS, YS, and E_b_ are shown for the tests performed in the longitudinal and transverse directions with respect to the extrusion direction. The CG sample had a UTS of approximately 530 MPa and a YS of 460 MPa. Slightly higher values were obtained in the longitudinal direction. Moreover, E_b_ was approximately 4% larger in the longitudinal direction than it was in the transverse direction. Following both hydrostatic extrusion and heat treatment, mechanical strength increases and elongation decreases. Noticeable differences were observed on the two measured directions. The highest value of UTS was obtained for the HE sample in the longitudinal direction; in the transverse direction, HE, HT1, and HT2 all displayed similar values. The value of YS gradually increased as the aging temperature increased. The opposite relationship existed between E_b_ and aging temperature. A noticeable difference in results obtained for the two measured directions could be observed. The values of all three parameters were larger in the longitudinal direction. For the CG sample, the differences were relatively small—approximately 10 MPa. However, the remaining samples displayed much larger differences, in the range of 80–140 MPa for YS and 60–100 MPa for UTS. The highest anisotropy of mechanical strength was observed for HE, while it was reduced after subsequent heat treatment. The work hardening capacity, which is defined as the ratio of UTS to YS, was the largest for the HE sample and gradually decreased for a higher aging temperature.

**Figure 9:**
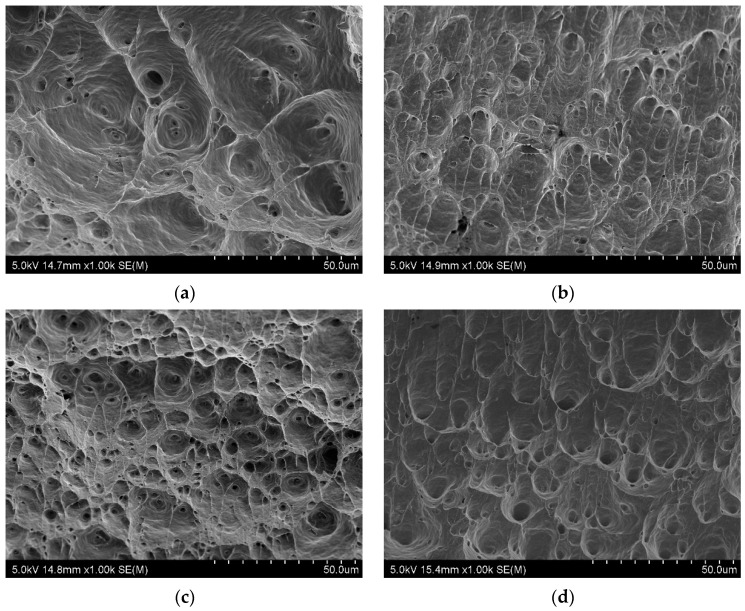
Fractures of the tensile samples cut from the transverse direction: (**a**) CG, (**b**) HE, (**c**) HT1 and (**d**) HT2.

The fracture surfaces of the samples are shown in Figure 9. Images were taken from the tensile specimens cut from the transverse direction in relation to the extrusion direction. In each case, the fracture was ductile and with a transcrystalline character. The main observed feature was the presence of numerous equiaxial dimples. The CG sample was larger than the samples after hydrostatic extrusion. In the case of the aging process, there was no correlation. This indicates that the size and subsequently the number of dimples were connected with grain size and dislocation density because these were the sites of the nucleation of the cavities, which may result in the formation of dimples. In [23], a decrease in grain size also led to a decrease in dimple size.

### 3.3. Corrosion

#### 3.3.1. Electrochemical Properties

**Figure 10:**
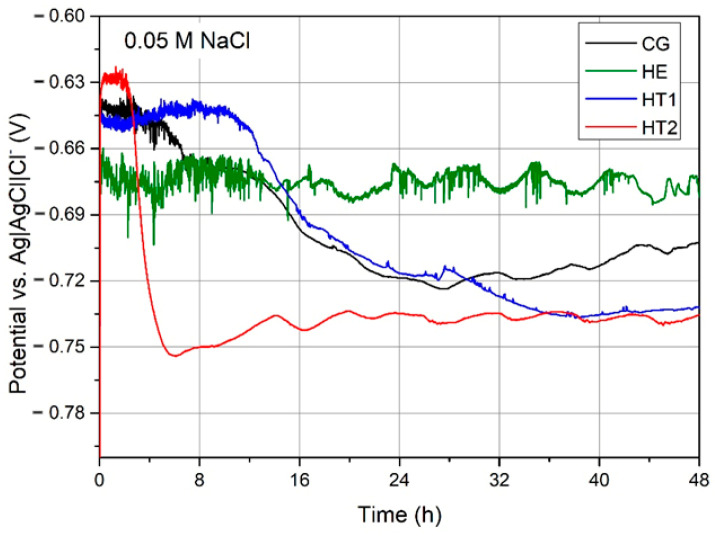
E_OCP_ over time for the CG, HE, HT1, and HT2 AA 7075 samples during 48 h immersion in 0.05 M NaCl.

Figure 10 shows the E_OCP_ over time for each sample during their 48 h immersion in 0.05 M NaCl. The values of E_OCP_ sharply increased, followed by a steady decrease over time. The only exception was the HE sample, which maintained a very unstable E_OCP_ level that oscillated between −0.70 and −0.67 V/Vref, producing an irregular line on the graph. For the HT2 sample, the E_OCP_ value stabilized at approximately −0.75 V/Vref, following 14 h of a sharp decrease. For the HT1 sample, the E_OCP_ value decreased steadily over almost 35 h before stabilizing at −0.72 V/Vref. Similarly, the E_OCP_ of the CG sample stabilized at −0.70 V/Vref following a gradual decrease. After 48 h, the HT2 sample displayed the lowest E_OCP_ value of approximately −0.75 V/Vref, and the HE sample displayed the highest value of approximately −0.67 V/Vref. 

**Figure 11:**
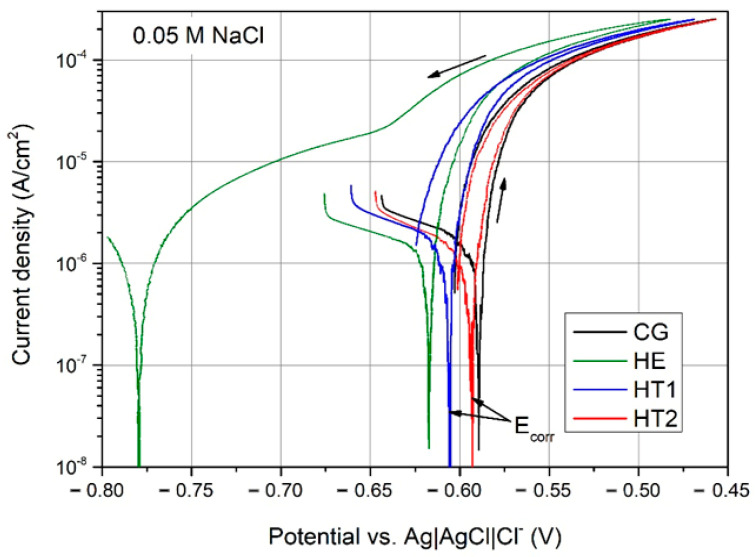
PP curves of the CG, HE, HT1, and HT2 AA 7075 samples following 600 s of E_OCP_ stabilization in 0.05 M NaCl solution.

Figure 11 shows representative cyclic PP curves during 600 s of E_OCP_ stabilization in 0.05 M NaCl solution. The electrochemical data obtained from the curves are listed in Table 2. During the forward scan, the shape of each curve was similar: a rapid increase in current density once corrosion potential (E_corr_) had been reached. This indicates that the anodic behavior was dominated by active dissolution. The cathodic current density during the forward scan was the lowest for the HE sample, higher for the aged HT1 and HT2 samples, and highest for the CG sample. This may indicate the acceleration of the oxygen reduction reaction. The b factor, which describes the inclination of the cathodic branch, was the highest for the CG sample and decreased with increasing aging temperature. The lowest E_corr_ was observed for the HE sample. This shifted to more anodic values following aging. Moreover, artificial aging lowers the corrosion current density (i_corr_), which was the lowest for the HT2 sample. This indicates that artificial aging at elevated temperatures reduces susceptibility to localized attacks. During the reverse scan, the HE curve significantly differed in shape. The hysteresis loops and repassivation potentials were somewhat similar for the aged and recrystallized samples. A large hysteresis and much lower repassivation potential (−780 mV/Vref) were recorded for the HE sample. This suggests differences in repassivation, and consequently in the morphology of the corrosion attack [24].

A comparison of the results of PP to the literature data for AA7075 shows that a similar course of the curves was observed as that in [25]; however, due to the lower pH of the 3.5% NaCl solution, the observed PP curves were shifted to less noble values, and a breakage potential was also observed. Similar findings in the case of E_corr_ were observed for AA7075 examined in 3.5% NaCl [26], but the values of i_corr_ were in the same range as that in the present study. An abrupt increase in a current density was observed here after reaching E_corr_, which indicated rapid dissolution. AA7075 was also investigated in [27], where 0.001 M NaCl was used for corrosion tests. The obtained average values of E_corr_ were higher for about 50–100 mV, but the scattering of the results was more pronounced in comparison to the results obtained in the present study. Similar values of i_corr_ were also observed.

#### 3.3.2. Postcorrosion Morphology

**Figure 12:**
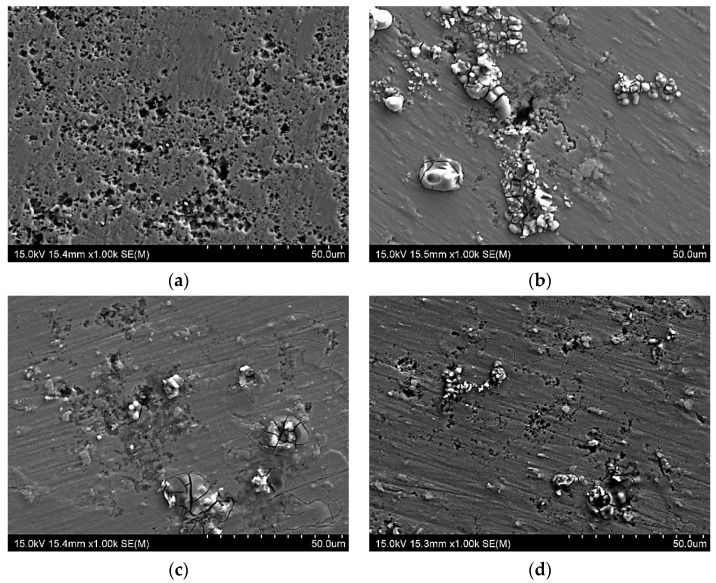
Sample surfaces following potentiodynamic polarization in 0.05 M NaCl: (**a**) CG, (**b**) HE, (**c**) HT1, and (**d**) HT2.

**Figure 13:**
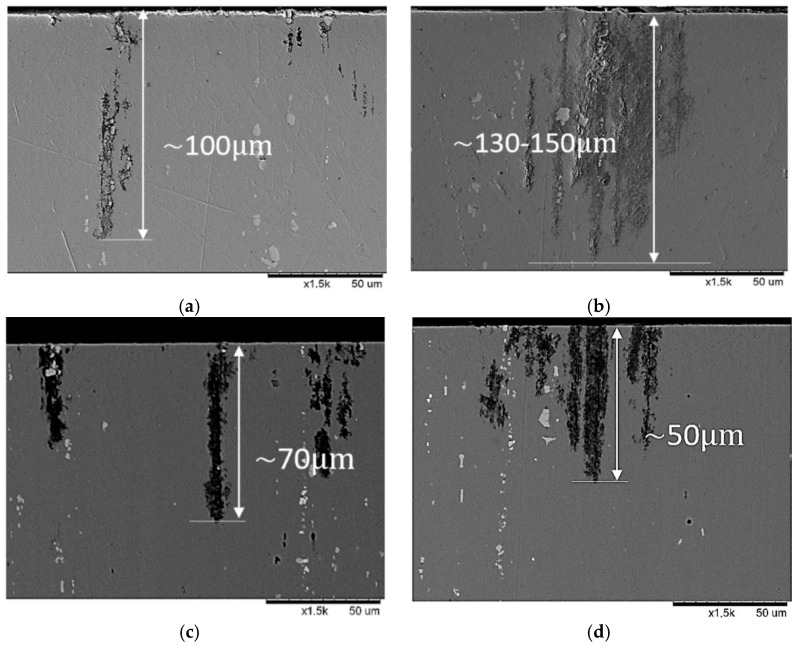
Sample cross-sections following potentiodynamic polarization in 0.05 M NaCl: (**a**) CG, (**b**) HE, (**c**) HT1, and (**d**) HT2.

Figure 12 presents the postcorrosion morphology following the PP tests. Corrosion products were detected on the surface of several samples. The most substantial differences between the samples were the depths and shapes of the pits rather than the number of pits present. Images of the cross-sections are shown in Figure 13. CG and HE samples had the deepest pits of approximately 100 and 130–150 μm, respectively. The HT1 and HT2 samples had the shallowest pits, of depth 50–70 μm. Although the pits were relatively narrow, for samples that had undergone hydrostatic extrusion were located in close proximity to one another, creating a network of pits and creating a larger region of degradation. The pit locations appeared to be correlated with grain shape and elongation in addition to the locations of the intermetallic particles. The hydrostatic extrusion process tends to align constituent particles into bands within the alloy, but no significant difference in their sizes was noted. At multiple locations, the pitting and initial corrosion damage was located close to the intermetallic inclusions, and propagated along the intermetallic particles. Moreover, the intermetallic particles were frequently observed to be intact, with corrosion occurring at the particle–matrix interface, resulting in the dissolution of the adjacent material (matrix).

## 4. Discussion

### 4.1. Microstructural Evolution through Hydrostatic Extrusion with Subsequent Aging

Microscopic observations of the material following hydrostatic extrusion reveal that the microstructure was severely deformed, with the grains significantly elongated (fibrous) along the extrusion direction. Microstructural evolution in Al during hydrostatic extrusion had been demonstrated [28] with three presented mechanisms of HAGB formation. The first mechanism is based on the formation of new grains as a result of dynamic recrystallization, the second describes continuous grain rotation as a result of mobile dislocation absorption into grain boundaries, and the third concerns neighbor switching due to slip banding. In general, the fraction of HAGBs increases with increasing strain. In this study, an applied equivalent strain was estimated at ε = 1.4. This relatively small strain resulted in the formation of a high density of dislocations. Higher strains would reduce the dislocation density and increase the misorientation angles of the grain boundaries. EBSD analysis showed that the samples had reached an average grain size of approximately 1.3 μm on the transverse plane following hydrostatic extrusion. In comparison, the reference sample displayed an average grain size of 17 μm. This shows that hydrostatic extrusion is an efficient means of grain refinement and, at this strain level, the formation of subgrain structures. The EBSD investigation further revealed that hydrostatic extrusion significantly changed the characteristics of the grain boundaries, increasing the fraction of LAGBs within the microstructure to 76%. The high fraction of LAGBs was caused by relatively low accumulated deformation. Previous work showed that applying hydrostatic extrusion to an Al–Mg–Si alloy at a similar level as that in the present study could further accommodate plastic strain via the dislocation of cells [29]. Further deformation would increase the misorientation angle between cells to above 15°, causing the formation of HAGBs. 

Grain orientation analysis provided information about the favorable crystallographic orientations within the microstructure following hydrostatic extrusion. The grains aggregated into two dominant orientations: <001> and <111>. The <111> orientation is typical for a fiber texture, whereas the <001> orientation may result from dynamic recrystallization caused by the temperature increase during hydrostatic extrusion [28]. As was shown, the grain orientation stimulates the deformation substructure: for grains with the <111> orientation, dense dislocation walls are formed; for grains with the <001> orientation, stable cell dislocation structures can be observed [30]. In contrast, within the CG sample, no clear texture or preferred crystallographic orientation was identified. This may have been caused by the solution annealing performed after the hydrostatic extrusion, leading to grain recrystallization following plastic deformation. 

In addition to grain size and texture, precipitates were also investigated. XRD studies showed that MgZn_2_ precipitates formed within the structure as a result of the aging process. In addition, η’ phases were observed by the TEM. An increase in aging temperature increased the size of the MgZn_2_ precipitates within the microstructure. Hydrostatic extrusion also influenced the precipitation phenomena. The work investigating an Al–Mg–Si alloy showed that a heterogeneous microstructure following hydrostatic extrusion results in differences in the precipitate sequence [30]. This is caused by thermal shocks during the deformation process, which may induce the formation of clusters before the precipitate forms. For pure Al, adiabatic heating during hydrostatic extrusion with a strain of ε = 1.4 should be approximately 120–130 °C, and may influence or initiate the precipitation process in the case of age-hardenable alloys [31]. For AA 7475, the hydrostatic extrusion process changed the precipitation kinetics and phase stability induced by the grain boundaries [32]. Precipitation occurs along fast diffusion paths, such as grain boundaries and dislocations. Therefore, a larger number of precipitates at grain boundaries was observed within a deformed microstructure, as in this study. However, due to the higher number of nucleation sites, the precipitates were smaller than those in coarse-grained material. This effect was most noticeable within the grain interiors. Nevertheless, the increase in temperature of artificial aging led to an increase in the size of the precipitates.

### 4.2. Influence of Microstructural Evolution on Mechanical Properties

The evaluation of the mechanical properties revealed that hydrostatic extrusion caused an increase in mechanical strength. The highest UTS (approximately 675 MPa) was obtained for the HE sample in the longitudinal direction. In the transverse direction, the HE, HT1, and HT2 samples demonstrated similar values (approximately 575 MPa) and greater than those the CG sample. The increase in aging temperature caused an increase in YS. The average tensile strength of the commercially available coarse-grained AA 7075 in a T6 state is approximately 570 MPa, with a YS of 500 MPa and an elongation at break of approximately 9% [33]. This indicates that the combination of hydrostatic extrusion and T6 thermal treatment increased tensile strength by up to 18% when compared with the heat treatment of CG AA 7075. 

Hydrostatic extrusion significantly increases mechanical strength, which is caused by the introduction of a considerable number of structural defects that inhibit the motion of dislocations during deformation. In the case of pure metals, when these defects are the only strengthening mechanism, the increase in mechanical strength is dependent on the value of true strain [34]. Nevertheless, in the case of age-hardened alloys such as AA 7075, strengthening from precipitates must also be considered. Following hydrostatic extrusion, and with dislocation slip in the dominant deformation mechanism, the particle strengthening of AA 7475 is limited by both enhanced grain boundary precipitation that does not contribute to an increase in mechanical strength, and the smaller size of precipitates within grain interiors, which are stronger barriers for moving dislocations [35]. Precipitates within grain interiors were also larger for the CG sample (see Figure 4) than for samples that had undergone hydrostatic extrusion. Therefore, precipitation strengthening is less effective for UFG material, with a larger contribution by structural defects such as grain boundaries and dislocations.

The anisotropy of mechanical properties was also investigated, as tensile tests were performed on samples cut from two directions, transverse and longitudinal, with respect to the extrusion direction. The microstructural anisotropy was significant as grains form fibrous shapes along the extrusion direction. This caused long HAGBs with interior LAGBs to create a network of subgrains. Regardless of the deformation ratio during hydrostatic extrusion, the AA 6060 alloy was characterized by weaker strength in the cross-section perpendicular to the extrusion direction [36], as was found in the present study for AA 7075. The anisotropy of AA 6060 increased with an increase in ε UTS and YS values indicated approximately 10% anisotropy, with higher values in the longitudinal direction for ε = 2.28. The anisotropy of mechanical properties was caused by the strong crystallographic macrotexture that develops in the material during hydrostatic extrusion. As a result, the spacing between HAGBs was smaller in the longitudinal plane than that in the transverse plane (see Figure 1). This caused a higher volume of HAGBs, which are stronger barriers for moving dislocations [34,37]. As a result, the samples cut in the longitudinal direction possessed higher mechanical strength.

### 4.3. Influence of Microstructural Evolution on Corrosion Resistance

The obtained results of the CG and HE samples show that the plastic deformation increased the corrosion susceptibility of the material. This may have resulted from the grain boundary characteristics (the fraction of LAGBs and HAGBs) of the samples being modified by the HE process. EBSD examination confirmed that HE contained a much larger fraction of LAGBs in comparison to CG. However, the absolute number and length of HAGBs were also greater. The dissolution of the material preferably occurred along HAGBs, since the HAGBs indicate regions with elevated levels of excess energy [38]. This phenomenon may have been the principal reason for the significantly lower corrosion resistance of the HE sample.

Postcorrosion analysis of the pit morphologies showed that, for CG, the pits were relatively narrow; for the samples that had undergone hydrostatic extrusion, the pits tended to develop inside the material, creating larger networks of dissolved material. Simultaneously, the depth of the corrosion pits decreased for samples that had been subjected to artificial aging. As the aging conditions and hydrostatic extrusion did not change the composition and size of the Al–Cu–Fe particles, the obtained results must be connected with differences in the matrix composition due to the growth of precipitates. In particular, the naturally aged HE sample exhibited more active breakdown potential than that of the peak-aged T6 HT2 sample, because the former had a higher potential difference between the intermetallic particles and the matrix and thus exhibited a stronger galvanic relationship [37]. The improvement of corrosion resistance following artificial aging can be attributed to the number of precipitates, as artificial aging significantly accelerates the precipitation effect. That is, the matrix composition of the material changes with aging due to precipitation, rendering it nobler and improving the corrosion resistance of the material. This may explain why the naturally aged HE was much more prone to dissolution and more susceptible to corrosion when compared to the artificially aged samples. Furthermore, artificial aging at higher temperatures leads to the coarsening of large precipitates at the expense of smaller precipitates, which in turn reduces the number of the precipitates within the microstructure. This effect may influence the overall corrosion behavior of the material, since corrosion is centered on specific sites in the heterogeneous microstructure, such as precipitates and intermetallic particles. Therefore, their limited number reduced the number of preferential nucleation sites of a corrosion attack [39]. 

## 5. Conclusions

In this paper, the effect of combined hydrostatic extrusion processing and various thermal treatments on the corrosion and mechanical properties of AA 7075 was investigated. Due to preserving the size of intermetallic particles that significantly influence the corrosion resistance, other microstructural components and their influence could be examined. The main conclusions from the present study are as follows:

- Hydrostatic extrusion with an equivalent strain of ε = 1.4 resulted in grain refinement from 17 to 1.3 µm. A microstructure consisting of fibrous grains oriented in the <001> and <111> directions with a fraction of LAGBs of 76% was obtained.
- As a result of thermomechanical treatment, an increase in tensile strength of up to 100 MPa was obtained when compared to the CG T6 state. Hydrostatic extrusion caused a noticeable anisotropy of the mechanical properties, as differences in tensile strength between longitudinal and transverse directions were in the range of 60–100 MPa.
- Artificial aging caused an increase in the size of MgZn_2_ precipitates within the microstructure, and led to improved corrosion resistance due to changes in the chemical composition of the matrix. The differences in mechanical strength in relation to the aging temperature were not significant due to the location of precipitates at the grain boundaries, which were not effective in strengthening the material.
- The optimal correlation of high mechanical strength and high corrosion resistance was obtained for the sample subjected to hydrostatic extrusion and subsequently aged at 120 °C for 24 h, corresponding to a T6 state.

## Figures and Tables

**Table 1:** Chemical composition of AA 7075.

Element	Zn	Mg	Cu	Cr	Ti	Si	Fe	Mn	Al
Content (wt. %)	5.70	2.40	1.50	0.19	0.04	0.09	0.23	0.06	balanced

**Table 2:** Average values of corrosion potential (E_corr_), corrosion current density (i_corr_), and repassivation potential (E_rep_), b factor calculated from the potentiodynamic polarization curves.

Sample	E_corr_, mV/Ref	i_corr_, µA/cm^2^	E_rep_, mV	b Factor, V/dec
CG	−590 ± 5	0.72 ± 0.08	−599 ± 3	0.048
HE	−615 ± 4	0.78 ± 0.13	−780 ± 3	0.039
HT1	−604 ± 4	0.66± 0.21	−617 ± 6	0.024
HT2	−592 ± 3	0.50 ± 0.05	−600 ± 1	0.024

## Data Availability

The data presented in this study are available on request from the corresponding author.
